# A New Index Based on Serum Creatinine and Cystatin C Can Predict the Risks of Sarcopenia, Falls and Fractures in Old Patients with Low Bone Mineral Density

**DOI:** 10.3390/nu14235020

**Published:** 2022-11-25

**Authors:** Jiaying Ge, Jiangping Zeng, Huihui Ma, Siqi Sun, Zheng Zhao, Yujie Jing, Chunhua Qian, Zhaoliang Fei, Ran Cui, Shen Qu, Ge Zhang, Hui Sheng

**Affiliations:** 1 Department of Endocrinology and Metabolism, Shanghai Tenth People’s Hospital, School of Medicine, Tongji University, Shanghai 200072, China; 2 Law Sau Fai Institute for Advancing Translational Medicine in Bone and Joint Diseases, School of Chinese Medicine, Hong Kong Baptist University, Kowloon Tsai, Hong Kong 999077, China

**Keywords:** low bone mineral density, sarcopenia, creatinine, cystatin C, fall, fracture

## Abstract

As new screening tools for sarcopenia, the serum sarcopenia index (SI) and creatinine/cystatin C ratio (CCR) had not been confirmd in a population with a high fragility fracture risk. This study aimed to evaluate whether SI and CCR indicators are useful for diagnosing sarcopenia and to determine their prediction values for future falls and fractures. A total of 404 hospitalized older adults were enrolled in this longitudinal follow-up study (mean age = 66.43 ± 6.80 years). The receiver operating curve (ROC) was used to assess the diagnostic accuracy of SI and CCR. Backward-selection binary logistic regression was applied to develop the optimal models for the diagnosis of new falls and fractures. SI had a significantly higher area under the curve (AUC) than CCR for predicting sarcopenia. The optimal models had acceptable discriminative powers for predicting new falls and fractures. Lower SI and CCR are the independent risks for sarcopenia, new falls, and fractures in the low-BMD population. SI and CCR, as easily accessible biochemical markers, may be useful in the detection of sarcopenia and in predicting the occurrence of new falls and fractures in patients with low BMD who have not previously experienced falls or fractures. However, further external validations are required.

## 1. Introduction

Sarcopenia is one of the important contributors to fragility fractures in the low bone mineral density (BMD) population, for it increases the risk of falls. According to large clinical RCT trials, bone-targeted anti-fracture therapies are only able to reduce the hip fracture risk by less than 50% with long-term treatment [1,2]. One of the key factors behind this is sarcopenia, which increases the risk of falls and then fractures. The morbidity of sarcopenia accounts for approximately 50% of osteopenia/osteoporosis patients [3,4]. As the population ages, the prevalence of osteoporosis and sarcopenia is expected to rise in the future [5,6]. Consequently, an anti-fracture strategy must pay more attention to sarcopenia screening and intervention during anti-osteoporosis therapy. However, there are no verified serum biomarkers for sarcopenia screening and fall/fracture prediction in patients with low BMD.

All creatine in the body is present in skeletal muscle, and the amount of creatine and the rate of creatine catabolism per unit of skeletal muscle mass are consistent [7,8]. Additionally, cystatin C (CysC) is derived from all nucleated cells, and CysC is completely reabsorbed and catabolized within the proximal tubule, regardless of age, sex, inflammation, malignancy, infection, or muscle mass. Thus, it is regarded as a reliable marker of kidney function [9,10]. Based on these characteristics, muscle mass can be further predicted by dividing the serum creatinine level by the serum CysC level. Due to the properties of free glomerular filtration and renal tubular reabsorption, timed urinary creatinine excretion is a reliable marker for predicting muscle mass [11]. Additionally, a surrogate for 24 h filtered creatinine has been proposed, which is calculated as the product of serum creatinine and glomerular filtration rate [12].

The study hypothesis is that CCR (creatinine/cystatin C ratio), or the SI (sarcopenia index) may predict sarcopenia, falls, and further fractures in a population with a high fragility fracture risk. This study aimed to evaluate and validate whether SI and CCR indicators can be used to diagnose sarcopenia and to investigate their predictive value for new falls and fractures in a low-BMD population.

## 2. Materials and Methods

### 2.1. Study Design and Participants

This study was conducted at the Department of Endocrinology and Metabolism at Shanghai Tenth People’s Hospital from November 2020 to March 2022. Patients aged 55 years or older and meeting any of the following criteria were included in this study: (1) *t*-score < −1 at any of the lumbar spine, hip, and femoral neck; (2) serum creatinine < 2.0 mg/L; (3) muscle strength, muscle mass, and physical performance data unavailable. Exclusion criteria were also applied: (1) severe organ failure (heart, respiratory and liver failure) or severe renal function decline (estimated glomerular filtration rate (eGFR) < 45 mL/min/1.73 m^2^); (2) severe systemic diseases (hematological disorder, systemic connective tissue disease, and malignant tumors); (3) mental illness or physical disability; (4) use of systemic steroids or immunosuppressants; (5) serum creatinine and cystatin C data not available. Two experienced clinicians jointly diagnosed the diseases. Data on muscle strength, physical performance, and muscle mass were obtained retrospectively from the medical record performed by the doctor at the initial visit. This research was approved by the ethics committee of Shanghai Tenth People’s Hospital (22K177), and the requirement for informed consent was waived due to the design of the study. All patients were given oral informed consent notification during the telephone follow-up.

### 2.2. Follow-Up Method

Researchers contacted participants or family members living with them via phone for follow-up visits to determine whether they had fallen or fractured a bone during the one-year study period. Follow-up by telephone or text message is a routine procedure, which is documented in the patient’s admission notification form and signed by the patient or his family. The researcher informed the participants of the purpose and nature of the study, the duration of participation, a description of the procedures, a description of foreseeable risks, disclosure of alternative treatments, and declaration of confidentiality of the study during the telephone contact. We then obtained the consent of the patient or his family again. In total, 18 patients were lost to follow-up because they or their families were unable to be contacted by telephone. Fall was defined as an unintentional fall to the ground, floor, or another lower level [13]. Fragility fracture was characterized by a fall from standing height or less or trauma that cannot result in a fracture for a healthy individual [14]. A new fall was defined as one or more new falls that occurred during the telephone follow-up after one year. One or more new fractures was marked as a new fracture.

### 2.3. Sarcopenia and Bone Mineral Density Assessments

Based on the Asian Working Group for sarcopenia 2019, sarcopenia is defined as low muscle mass combined with low muscle strength or low physical performance [15]. Assessment of sarcopenia was completed at initial recruitment.

Muscle mass and bone mineral density were measured by dual-energy X-ray absorptiometry (DXA) scanner (Hologic Discovery QDR Series, Bedford, MA, USA). Low muscle mass was defined as ASMI (appendicular skeletal muscle index, a ratio of appendicular muscle mass to squared height) less than 7.0 kg/m^2^ for men or 5.4 kg/m^2^ for women. Low BMD was defined as BMD > 1 standard deviations below healthy adults of the same sex and race (*t*-score < −1) [16]. If hip, lumbar spine (L1~L4), or femoral neck BMD met criteria, low BMD was diagnosed. T scores were calculated using the DXA database.

Calf circumference (CC) was measured bilaterally with a non-elastic dipstick. The minimum circumference was then recorded.

Hand grip strength (HGS) was assessed with a strain guage sensor-based handheld digital dynamometer (CAMRY EH10, Xiangshan, China). The strength of both hands was measured twice, and the highest value of either was recorded. Low muscle strength was defined as HGS of less than 28 kg for men or 18 kg for women.

Physical performance was evaluated by five chair stand test (FCST). Participants were asked to cross their arms in front of their chest and perform five chair stands as soon as possible. Time was recorded (in seconds) from the start sitting position to the final standing position on the fifth stage. Low physical performance was defined as FCST of greater than 12 s.

### 2.4. Patients’ Information and Laboratory Tests

Patients information and laboratory tests were both obtained during the initial recruitment. As part of the electronic medical record, basic information and comorbidities are collected, including type 2 diabetes mellitus (T2DM) and cardiovascular disease (CVD), and the history of smoking, drinking, fractures, and falls. Based on Controlling Nutritional Status (CONUT) Score [17], the nutrition state was categorized into four levels: normal (0–1 points), mild (2–4 points), mid (5–8 points), and severe (>8 points). Exercise state was assessed using metabolic equivalent (MET) calculated using the following formula: MET coefficient of activity × duration (hours) × frequency (days per week). Based on the MET coefficients, four levels of activity were defined, including sitting, walking, moderate activity, and vigorous activity, corresponding to MET coefficients of 1.0, 3.3, 5.5, and 8.0, respectively [18].

After an 8 h fast, blood samples from all participants were obtained by trained nurses early in the morning of the second day after admission and then transferred to the clinical laboratory of our hospital. Laboratory parameters measured included bone metabolism indexes (25-hydroxy-vitamin D (25(OH)D), calcitonin, osteocalcin, C-terminal telopeptide of beta-I collagen (beta-CTX), parathyroid hormone (PTH), renal function indexes (creatinine, CysC, eGFR), 24 h urine creatinine excretion (24 h Ucr)), lipid metabolism-related index (total cholesterol), and glycosylated hemoglobin (HbA1c).

Serum creatinine levels were measured using the Jaffe method (Architect Alinity c). Serum CysC levels were measured using a latex agglutination turbidimetric immunoassay (Architect c16000). The eGFR_CysC_ was calculated based on CysC using the following equation [19]: if serum CysC ≤ 0.8, eGFR_CysC_ = 133 × (CysC/0.8) ^−0.499^ × 0.996^Age^ [×0.932 if female]; if serum CysC > 0.8, eGFR_CysC_ = 133 × (CysC/0.8) ^−1.328^ × 0.996^Age^ [×0.932 if female]. The CCR was calculated as serum creatinine/cystatin C, and the SI was calculated as serum creatinine × eGFR_CysC_.

### 2.5. Statistical Methods

Continuous variables were presented as mean ± standard deviation (SD) or median (interquartile range, IQR), and categorical variables as absolute and relative frequencies. The statistical significance of differences between two independent samples was evaluated using independent two-sample t-test or Mann–Whiney U test for continuous variables and Chi-square test or Fisher exact test for categorical variables. Spearman’s correlation analysis was performed to assess the correlation. Binary logistic regression analysis was performed to identify the independent association of sarcopenia with SI and CCR, in which we adjusted for confounders based on literature and expert knowledge. Furthermore, the area under the curve (AUC) was applied to evaluate the diagnostic efficacy of serum SI and CCR levels in detecting sarcopenia. Comparisons between receiver operating characteristic (ROC) curves were performed using the Delong method [20]. We established optimal models for new fractures and falls within one year using multivariable binary logistic regression analyses with backward selection. For the models using binary logistic regression, z-scores of SI and CCR were included as continuous variables to calculate the odds ratios (OR) per 1-SD increase with 95% confidence intervals (CIs). Additionally, ROC curve analyses were performed to evaluate the diagnostic value of the optimal models. Statistical analysis was conducted in SPSS 20.0 and MedCalc Statistical Software 20.112. All statistical tests were two-sided, and statistical significance was identified based on a *p*-value of <0.05.

## 3. Results

### 3.1. Baseline Characteristics of Participants Based on the Presence or Absence of Sarcopenia

Five-hundred and thirty-six inpatients were assessed for their eligibility for inclusion, and 132 patients were excluded based on exclusion criteria. As a result, a total of 404 patients (258 women and 146 men; mean age: 66.43 ± 6.80 years) were included in the study (Figure S1).

The baseline characteristics of the study population according to the presence of sarcopenia were summarized in Table 1. Compared to patients without sarcopenia, patients with sarcopenia were older; had lower ASMI, CC, HGS, MET, nutrition, and 24 h Ucr values compared to non-sarcopenia patients; but had higher values for body fat percentage, FCST, CysC, and incidence of prior fracture. There were no differences in sex, body mass index (BMI), spine BMD, 25(OH)D, calcitonin, osteocalcin, beta-CTX, PTH, HbA1c, total cholesterol, or serum creatinine. Notably, those with sarcopenia had significantly lower CCR and SI (both *p* < 0.001).

### 3.2. Associations of SI and CCR with CC, HGS, FCST, ASMI, and BMD

**Figure 1:**
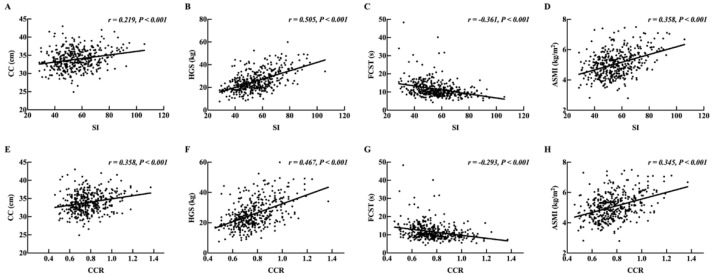
(**A**–**D**) Simple correlation analysis of CC, HGS, FCST, and ASMI with SI. (**E**–**H**) Simple correlation analysis of CC, HGS, FCST, and ASMI with CCR. SI: sarcopenia index; CC: calf circumference; HGS: hand grip strength; FCST: five chair stand test; ASMI: appendicular skeletal muscle index; CCR: creatinine/cystatin C ratio.

As shown in Figure 1, we investigated the relationships of SI and CCR with CC, HGS, FCST, and ASMI. Calf circumference, a simple indicator to screen sarcopenia, showed a significant positive correlation with both SI and CCR. In addition, HGS, FCST, and ASMI, diagnostic criteria for sarcopenia, were statistically associated with SI and CCR. Clearly, the correlations between SI and HGS (r = 0.505, *p* < 0.001), FCST (r = −0.361, *p* < 0.001), and ASMI (r = 0.358, *p* < 0.001) were stronger than the correlations between CCR and HGS (r = 0.467, *p* < 0.001), FCST (r = −0.293, *p* < 0.001), and ASMI (r = 0.345, *p* < 0.001), whereas SI’s correlation with CC (r = 0.219, *p* < 0.001) was lower than CCR’s (r = 0.358, *p* < 0.001). Additionally, significant correlations were found between both SI and CCR and different sites of BMD (Figure S2).

### 3.3. SI and CCR Were Independent Predictive Factors for Sarcopenia

The effects of SI and CCR on the risk of sarcopenia have been investigated using logistic regression analyses, as presented in Table 2. In the univariate model, SI and CCR both were significantly associated with the risk of sarcopenia (OR per 1-SD increase 0.537, 95% CI 0.425–0.680; OR per 1-SD increase 0.645, 95% CI 0.516–0.805), the results of which were sustained after extensive adjustment for potential confounders, including age, sex, BMI, MET, nutrition, history of alcohol and smoking, T2DM, cardiovascular disease (CVD), history of fractures and falls, and minimum *t*-score (Min *t*-score).

To further assess whether SI and CCR are valuable predictors for sarcopenia, the DeLong test was used (Figure S3). The AUCs for serum SI and CCR were 0.677 (95% CI 0.629–0.722) and 0.638 (95% CI 0.589–0.684), respectively. The AUC of SI was significantly larger than that of CCR (*p* < 0.001).

### 3.4. Lower Serum SI and CCR Levels Were Both Independently Associated with the Occurrence of New Fall and New Fracture within One Year

Multivariate logistic regression analyses were performed to analyze the associations between SI and CCR and a new fall and a new fracture within one year. Table 3 and Table S1 indicate that in the baseline non-fall population (*n* = 378), SI and CCR were inversely associated with new falls within one year (OR per 1-SD increase 0.472, 95% CI 0.275–0.810, *p* < 0.01; OR per 1-SD increase 0.507, 95% CI 0.302–0.849, *p* < 0.05) in the multivariable-adjusted model (Model 3) (age, BMI, sex, Min *t*-score and prior fracture). In the non-fracture population at baseline (*n* = 293), SI was inversely associated with new fractures within one year (OR per 1-SD increase 0.432, 95% CI 0.192–0.971, *p* = 0.042) in the multivariable-adjusted model (Model 3) (age, BMI, sex, Min *t*-score, and prior fall). However, CCR was no longer significantly associated with new fractures within one year after adjustment for the same variables.

We found that SI, CCR, BMI, Min *t*-score, and prior fracture are independent predictors of new falls in a non-fall population. Meanwhile, SI, CCR, Min *t*-score, and prior fall are independent predictors of new fractures in the non-fracture population.

### 3.5. Diagnostic Value of SI, CCR, and Predictive Equations for New Fall and New Fracture within One Year

All covariates in Model 3 (age, BMI, sex, Min *t*-score, prior fall, or prior fracture) were analyzed in multivariate binary logistic regression analyses with backward selection, resulting in the elimination of some covariates. For participants without prior fall, SI, BMI, prior fracture, and Min *t*-score were the optimal models for a new fall: logit (P1) = 5.043–0.054 × SI—0.253 × BMI (kg/m^2^)—1.328 × prior fracture (yes = 1)—0.576 × Min *t*-score. For participants without a fracture at baseline, SI, prior fall, and Min *t*-score were the optimal models for a new fracture: logit (P2) = −3.312–0.048 × SI + 2.009 × prior fall (yes = 1) −1.003 × Min *t*-score. In repeating the above analyses for CCR, for participants without a prior fall, CCR, BMI, prior fracture, and Min *t*-score were the optimal models for a new fall: logit (P3) = 5.360–4.639 × CCR–0.239 × BMI (kg/m^2^)—1.309 × prior fracture (yes = 1)—0.560 × Min *t*-score. For participants without a fracture at baseline, CCR, prior fall, and Min *t*-score were the optimal models for a new fracture: logit (P4) = −2.771–4.119 × CCR + 2.055 × prior fall (yes = 1)—0.986 × Min *t*-score.

**Figure 2:**
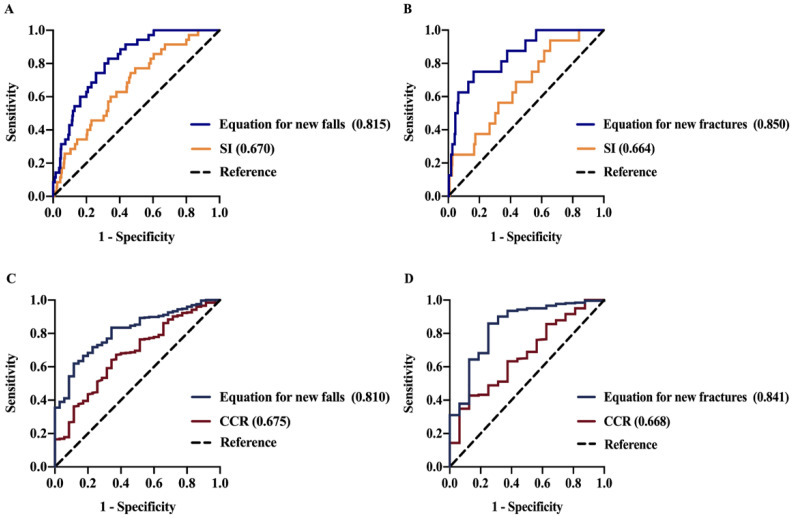
(**A**,**B**) ROC curves of SI and the equations (including SI) for new falls and new fractures within one year. (**C**,**D**) ROC curves of CCR and the equation (including CCR) on new falls and new fractures within one year. The reference is the tracing of the ROC analysis of the equation for predicting sarcopenia. Equation (including SI) for new falls: 5.043–0.054 × SI—0.253 × BMI (kg/m^2^)—1.328 × prior fractures (yes = 1)—0.576 × Min *t*-score. Equation (including SI) for new fractures: −3.312–0.048 × SI + 2.009 × prior falls (yes = 1)—1.003 × Min *t*-score. Equation (including CCR) for new falls: 5.360–4.639 × CCR–0.239 × BMI (kg/m^2^)—1.309 × prior fracture (yes = 1)—0.560 × Min *t*-score. Equation (including CCR) for new fractures: −2.771–4.119 × CCR + 2.055 × prior fall (yes = 1)–0.986 × Min *t*-score. Abbreviations: ROC: receiver operating characteristic; SI: sarcopenia index; CCR: creatinine/cystatin C ratio.

According to ROC curve analyses, the AUCs of logit (P1) and logit (P3) were 0.815 (95% CI 0.753–0.877) and 0.810 (95% CI 0.746–0.874) for predicting new falls in people without prior falls. Additionally, the AUC of logit (P2) and logit (P4) were 0.850 (95% CI 0.758–0.942) and 0.841 (95% CI 0.734–0.948) for predicting new fractures in those without previous fractures (Figure 2). In addition, the AUCs of the predictive equations that predict a new fracture and new fall were both significantly greater than those of SI or CCR alone, suggesting that the predictive equations were more reliable than SI and CCR alone. The cutoff value of logit (P1) was 0.078 with a sensitivity of 82.9% and specificity of 66.9%; that of logit (P2) was 0.083 with a sensitivity of 75% and specificity of 83.7%; that of logit (P3) was 0.066 with a sensitivity of 62% and specificity of 88.6%; that of logit (P4) was 0.094 with a sensitivity of 86% and specificity of 75% (Table S2).

## 4. Discussion

This study first showed that serum SI and CCR are independently associated with muscle strength, muscle mass, and physical performance in the elderly with low BMD. The correlation coefficients between SI and muscle strength, muscle mass, and physical performance demonstrated superior diagnostic value compared to CCR for sarcopenia. Furthermore, we found that decreased SI and CCR levels were independent risk factors for a new fall and a new fracture within a year of the study. Based on these findings, we developed predictive equations with a high diagnostic effectiveness for the prediction of new falls and fractures, equations (including SI) with AUCs of 0.815 and 0.850, and equations (including CCR) with AUCs of 0.810 and 0.841. 

Low BMD is well known to be associated with sarcopenia [21,22]. A Japanese study found that sarcopenia was responsible for 32–38% of osteopenia and 38.9–44.2% of osteoporosis in female patients over 60 [23]. Consistently, sarcopenia was prevalent in 38.4% of the participants in our study. BMD at three sites is considered an essential diagnostic criterion for osteoporosis. A previous study demonstrated that serum creatinine or creatinine clearance calculated using the Cockcroft–Gault equation significantly correlated with hip BMD [24]. SI and CCR were strongly correlated with BMD at three key sites in our study.

This study first demonstrated that CCR and SI can independently predict sarcopenia in patients with low BMD. Sarcopenia risk increases 1.463 times for each SD reduction in SI. The low SI level remained a risk factor for sarcopenia, even after adjusting the Min *t*-score. A significant advantage of SI over CCR is its diagnostic accuracy in predicting sarcopenia, indicating it could be more useful in screening sarcopenia in older patients with low BMD. CCR has been shown to be correlated positively with muscle mass and muscle strength [25,26,27]; however, to the best of our knowledge, no study has evaluated whether CCR is correlated with sarcopenia in patients with low BMD. Compared with CCR, this study showed that SI correlated better with muscle mass, muscle strength, and physical performance. Several studies, including those involving cancer patients, had demonstrated that SI was positively related to muscle mass and muscle strength [28,29]. In contrast, our disease was more prevalent than cancer due to a higher prevalence of low BMD overall. The association between SI and physical performance was rarely evaluated in previous studies. SI was reported to be positively correlated with gait speed [12], and our study found SI to be negatively correlated with FCST. This suggests that SI is significantly associated with physical performance, not just muscle mass and strength. 

This study examined the diagnostic efficacy of SI and CCR in predicting new falls or new fractures within one year for patients with low BMD. A fall or fracture may result in psychological effects, such as fear of fall or fracture, which may, in turn, lead to self-restricted activity levels, resulting in a decline in physical performance [30,31]. A feature of this study is that it excluded participants with a prior fracture or fall at baseline, thereby reducing the impact of a previous falls or fractures on outcomes. Some research indicated that osteopenia/osteoporosis negatively affects physical performance and independence in activities of daily living [32,33]. Falls are strongly associated with various components of sarcopenia, including decreased muscle strength and physical performance, and low BMD as well [34,35]. Moreover, low BMD may result in a decrease in muscle strength, which can further contribute to poor balance and fractures. This study showed that lower SI and CCR levels were independent risks of a new fall within one year in individuals who had not previously fallen at baseline. The risk of a new fall increased by 2.119 times for each SD reduction in SI, and by 1.972 times for each SD reduction in CCR, albeit adjusted for potential confounders. Most fractures occur as a result of falls, and up to 87% of fractures in the elderly are caused by falls [36,37]. In addition, lower SI and CCR levels were identified as independent risk factors for a new fracture within one year among those without a fracture at baseline. Low BMD was positively associated with the risk of fracture [38]. Despite adjustment for confounders including Min *t*-score, the risk of new fracture increased by 2.315-fold for each SD decrease in SI.

A study showed a positive correlation between calf circumference and ASMI, which can be used to diagnose sarcopenia [39]. As demonstrated in this study, SI and CCR were significantly correlated with CC, further confirming the correlation between SI and CCR and muscle mass. It has been suggested that muscle strength is more important than muscle mass and that HGS is more effective in predicting poor clinical outcomes [40]. We found a higher correlation between SI and HGS than ASMI, which demonstrated its utility in predicting adverse clinical outcomes. Muscle strength and physical performance may deteriorate with sedentary lifestyles; physical exercise mitigates age-related declines in muscle strength [41]. Our study found that lower levels of exercise were associated with the presence of sarcopenia.

Our study examined for the first time the diagnostic efficacy of CCR and SI in determining sarcopenia in patients with low BMD, and we proposed predictive equations to detect new falls and fractures within one year. It is also important to note that this study has several limitations. First, our study was a single-center analysis that lacks universality, so multicenter work to confirm our conclusion is required. Second, this was a non-validation study, and we need internal and external validation to ensure that SI, CCR, and the predictive equations are reliable for predicting sarcopenia, new fractures, and new falls. Third, non-renal factors other than skeletal muscle mass, such as inflammation and dietary protein intake, may affect creatinine or cystatin metabolism and influence its changes. Fourth, prior history of falls and fractures based on telephone questionnaires may reflect recall bias.

These findings demonstrate that serum SI outperforms the CCR in predicting sarcopenia in patients with low BMD, but both SI and CCR have high diagnostic value for predicting new falls and fractures. Therefore, future studies are needed to determine these findings. Since creatinine and CysC levels are usually measured in clinical practice, the utilization of these parameters may facilitate early sarcopenia detection and prevent further falls and fractures in patients with low BMD.

## Figures and Tables

**Table 1:** Differences in clinical characteristics between the non-sarcopenia and sarcopenia group.

Characteristics	Total (*n* = 404)	Non-Sarcopenia (*n* = 249)	Sarcopenia (*n* = 155)	*p*
Age, years	66 (10)	65 (10)	68 (9)	<0.001 ***
Gender (female), *n* (%)	258, 63.9	155, 62.2	103, 66.5	0.393
MET	1008 (1386)	1107 (1533)	661.5 (1386)	<0.001 ***
Nutrition				<0.001 **
Normal, *n* (%)	330, 81.7	215, 86.3	115, 74.2	
Mild, *n* (%)	70, 17.3	34, 13.7	36, 23.2	
Mid, *n* (%)	4, 1	0, 0	4, 2.6	
Severe, *n* (%)	0, 0	0, 0	0, 0	
Ever smoker, *n* (%)	71, 17.6	46, 18.5	25, 16.1	0.547
Ever drinker, *n* (%)	40, 9.9	26, 10.4	14, 9	0.645
Prior fall, *n* (%)	26, 6.4	11, 4.4	15, 9.6	0.036 *
Prior fracture, *n* (%)	111, 27.5	55, 22.1	56, 36.1	<0.01 **
T2DM, *n* (%)	276, 68.3	168, 67.5	108, 69.7	0.643
CVD, *n* (%)	114, 28.2	61, 24.5	53, 34.2	0.035 *
BMI, kg/m²	23.53 (4.24)	23.66 (4.29)	23.31 (4.48)	0.665
Calf circumference, cm	33.91 ± 2.76	34.14 ± 2.75	33.55 ± 2.75	0.042 *
Body fat percentage, %	37.9 (10)	36.75 (10.2)	39 (9.7)	0.017 *
HGS, kg	24 (11.6)	26.3 (8.69)	20.7 (7.32)	<0.001 ***
FCST, s	10.44 (4.31)	9.44 (2.41)	13.53 (5.54)	<0.001 ***
ASMI, kg/m²	5.06 ± 0.834	5.21 ± 0.89	4.82 ± 0.67	<0.001 ***
Spine BMD, g/cm²	0.819 (0.194)	0.823 (0.186)	0.807 (0.206)	0.539
Hip BMD, g/cm²	0.775 ± 0.122	0.793 ± 0.121	0.747 ± 0.118	<0.01 **
Femoral neck BMD, g/cm²	0.649 ± 0.107	0.664 ± 0.108	0.623 ± 0.1	<0.01 **
Min *t*-score	−2.5 (1.2)	−2.4 (1.3)	−2.5 (1.3)	0.165
25(OH)D, ng/mL	24.12 (16.51)	23.91 (15.51)	25.11 (17.93)	0.549
Calcitonin, pg/mL	5.93 (6.64)	5.91 (6.25)	5.98 (8.14)	0.162
Osteocalcin, ng/mL	12.52 (6.23)	12.85 (5.74)	12.12 (6.83)	0.32
Beta-CTX, ng/mL	0.35 (0.28)	0.36 (0.27)	0.33 (0.3)	0.311
PTH, pg/mL	29.4 (22.8)	29.8 (19.6)	29.3 (28.3)	0.67
HbA1c, %	7.42 (3.46)	7.32 (3.34)	7.47 (3.53)	0.612
Total cholesterol, mmol/L	4.29 (1.4)	4.28 (1.39)	4.31 (1.31)	0.842
Serum creatinine, mg/L	0.79 (0.21)	0.74 (0.18)	0.72 (0.26)	0.381
Serum cystatin C, mg/L	1.04 (0.27)	0.92 (0.25)	1.03 (0.38)	<0.001 ***
eGFR_CysC_	75.15 (29.7)	80.13 (27.01)	66.35 (29.93)	<0.001 ***
eGFR, mL/min/1.73 m^2^	91.1 (17.93)	92.64 (14.7)	88.09 (23.02)	<0.001 ***
24 h Ucr, mg/d ^a^	7.73 (3.75)	8.24 (3.87)	7.07 (3.27)	<0.001 ***
CCR	0.77 (0.19)	0.77 (0.18)	0.7 (0.16)	<0.001 ***
SI	54.12 (17.5)	57.3 (16.74)	48.59 (15.7)	<0.001 ***

Continuous variables with normal distribution are presented as mean ± SD, continuous variables with nonnormal distribution are presented as median (IQR), and categorical data are presented as *n* (%). Abbreviations: MET: metabolic equivalent; T2DM: type 2 diabetes mellitus; CVD: cardiovascular disease; BMI: body mass index; HGS: hand grip strength; FCST: five chair stand test; ASMI: appendicular skeletal muscle index; 25(OH)D: 25-hydroxy vitamin D; Beta-CTX: C-terminal telopeptide of beta-I collagen; PTH: parathyroid hormone; HbA1c: glycosylated hemoglobin; eGFR: estimated glomerular filtration rate; Ucr: urine creatinine excretion; CCR: creatinine/cystatin C ratio; SI: sarcopenia index; SD: standard deviation; IQR: interquartile range. ^a^ 24 h urine sample was available in 321 patients. * *p* < 0.05, ** *p* < 0.01, *** *p* < 0.001.

**Table 2:** Univariate and multivariable analyses for logistic regression of sarcopenia.

		Sarcopenia	
		OR (95%CI)	*p*
SI (per 1-SD) increase			
	Univariate	0.537 (0.425, 0.680)	<0.001 ***
	Model 1	0.574 (0.437, 0.755)	<0.001 ***
	Model 2	0.632 (0.476, 0.840)	<0.01 **
	Model 3	0.624 (0.465, 0.836)	<0.01 **
CCR (per 1-SD) increase			
	Univariate	0.645 (0.516, 0.805)	<0.001 ***
	Model 1	0.699 (0.547, 0.895)	<0.01 **
	Model 2	0.763 (0.590, 0.985)	<0.05 *
	Model 3	0.750 (0.576, 0.976)	<0.05 *

Univariate: univariate logistic regression analyses for the associations between SI and CCR and sarcopenia. Multivariate logistic regression analyses were performed to determine whether SI and CCR are independently associated with sarcopenia. Model 1: Adjusted for age and sex. Model 2: Model 1 additionally adjusted for BMI, MET, and nutrition. Model 3: Model 2 additionally adjusted for alcohol, smoking, type 2 diabetes mellitus, cardiovascular disease, history of falls and fractures, and Min *t*-score. Abbreviations: OR: odds ratio; 95% CI: 95% confidential interval. SI: sarcopenia index; CCR: creatinine/cystatin C ratio; BMI: body mass index; MET: metabolic equivalent; Min: minimum. Dependent variable: sarcopenia; independent variables: SI, CCR, age, sex, BMI, MET, nutrition, alcohol, smoking, type 2 diabetes mellitus, cardiovascular disease, history of falls and fractures and Min *t*-score. * *p* < 0.05, ** *p* < 0.01, *** *p* < 0.001.

**Table 3:** Multivariable analyses for logistic regression of new fall and new fracture within one year.

	Model 1		Model 2		Model 3	
	OR (95%CI)	*p* Value	OR (95%CI)	*p* Value	OR (95%CI)	*p* Value
New fall (N = 41) ^a^						
SI (per 1-SD)	0.485 (0.313, 0.751)	<0.01 **	0.495 (0.298, 0.823)	<0.01 **	0.472 (0.275, 0.810)	<0.01 **
Age (years)	-	-	1.007 (0.949, 1.069)	0.822	1.015 (0.956, 1.077)	0.629
BMI (kg/m^2^)	-	-	0.743 (0.642, 0.86)	<0.001 ***	0.772 (0.662, 0.902)	<0.01 **
Sex (female vs. male)	-	-	-	-	2.119 (0.794, 5.661)	0.134
Min *t*-score	-	-	-	-	0.473 (0.284, 0.787)	<0.01 **
Prior fracture	-	-	-	-	0.257 (0.088, 0.747)	0.013 *
Prior fall	-	-	-	-	-	-
New fracture (N = 37) ^b^						
SI (per 1-SD)	0.473 (0.256, 0.873)	0.017 *	0.392 (0.190, 0.812)	0.012 *	0.432 (0.192, 0.971)	0.042 *
Age (years)	-	-	0.961 (0.881, 1.047)	0.359	0.965 (0.874, 1.066)	0.485
BMI (kg/m^2^)	-	-	0.876 (0.734, 1.045)	0.142	0.962 (0.798, 1.159)	0.681
Sex (female vs. male)	-	-	-	-	1.38 (0.29, 6.562)	0.686
Min *t*-score	-	-	-	-	0.366 (0.185, 0.725)	<0.01 **
Prior fall	-	-	-	-	7.328 (1.599, 33.585)	0.01 *
Prior fracture	-	-	-	-	-	-

Multivariate logistic regression analyses were performed to determine whether SI was independently associated with new fall and new fracture within one year. Abbreviations: SI: sarcopenia index; SD: standard deviation; OR: odds ratio; 95% CI: 95% confidential interval; BMI: body mass index; Min: minimum. Dependent variables: new fracture and new fall; Independent variables: SI, age, BMI, gender, Min *t*-score, and history of falls and fractures. ^a^ Logistic regression to predict fall at 12 months’ follow-up in participants without a fall at baseline (*n* = 378). ^b^ Logistic regression to predict fracture at 12 months’ follow-up in participants without a fracture at baseline (*n* = 293). * *p* < 0.05, ** *p* < 0.01, *** *p* < 0.001.

## Data Availability

The data presented in the study are available within the article.

## Supplementary Materials

The following supporting information can be downloaded at: https://www.mdpi.com/article/10.3390/nu14235020/s1, Figure S1. Participants flow chart. Figure S2. (A–C) Simple correlation analyses of spine BMD (L1~L4), hip BMD and femoral neck BMD with SI. (D–F) Simple correlation analyses of spine BMD (L1~L4), hip BMD and femoral neck BMD with CCR. SI: sarcopenia index; CCR: creatinine/Cystatin C ratio. Figure S3. ROC curves of SI (A) and CCR (B) in predicting sarcopenia. Reference is the tracing of ROC analysis of equation in predicting sarcopenia. Abbreviations: ROC: Receiver Operating Characteristic; SI: sarcopenia index; CCR: creatinine/Cystatin C ratio. Table S1. Multivariable analyses for logistic regression of new fall and new fracture within one year. Table S2. ROCs for SI, CCR and predictive equations as predictors of new fall and new fracture.
