# Supplementary material for: A New Index Based on Serum Creatinine and Cystatin C Can Predict the Risks of Sarcopenia, Falls and Fractures in Old Patients with Low Bone Mineral Density
Source: Nutrients. 2022 Nov 25;14(23):5020. doi: 10.3390/nu14235020 (PMC9738634; doi:10.3390/nu14235020)
Supplement: Supplementary file 1 [file nutrients-14-05020-s001.zip › nutrients-1966607-supplementary.pdf]

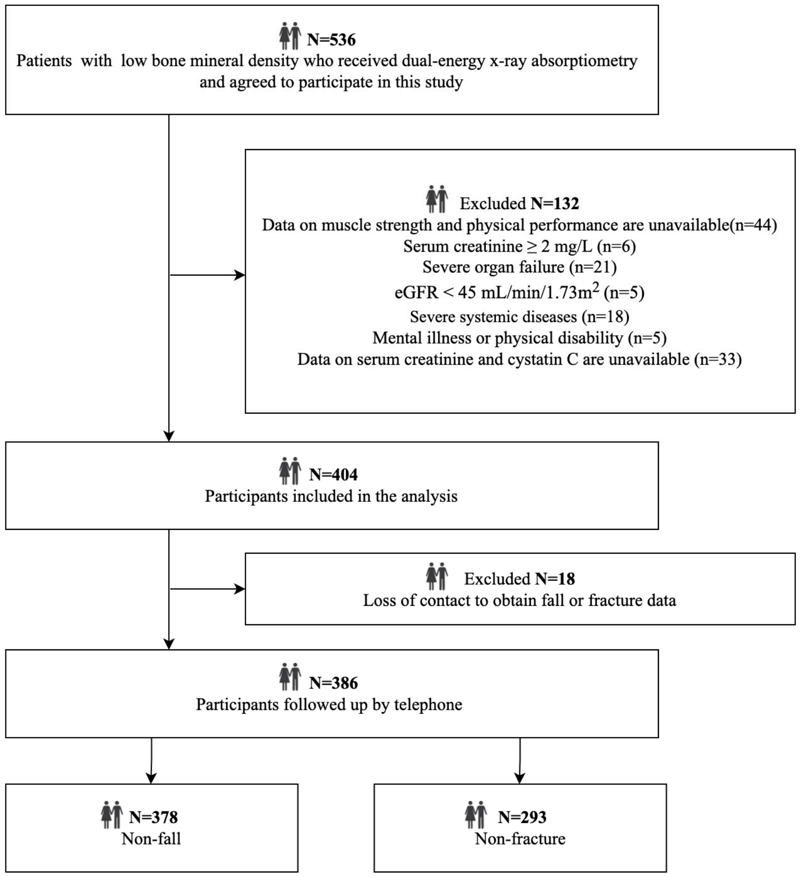

Figure S1. Participants flow chart.

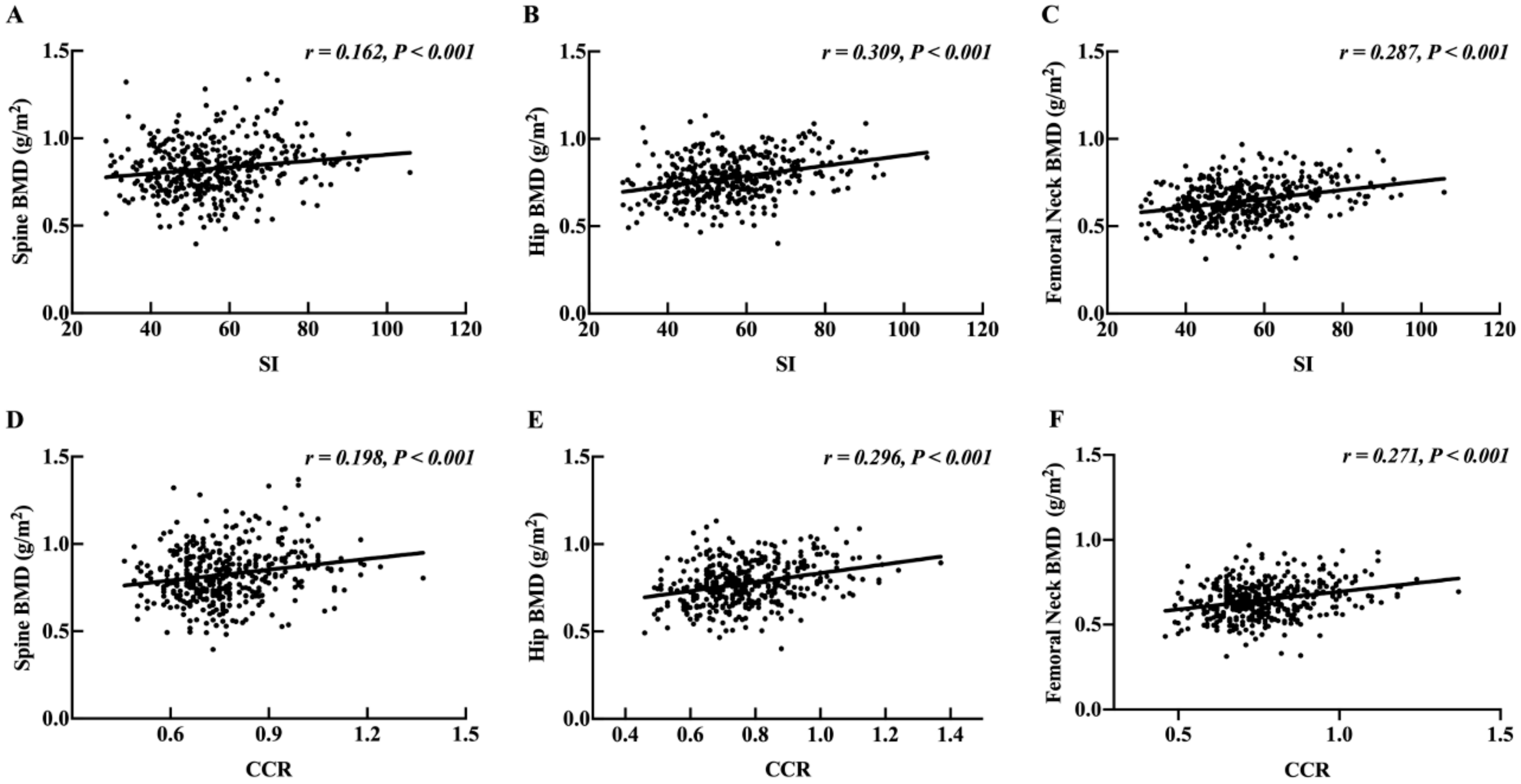

Figure S2. (A-C) Simple correlation analyses of spine BMD (L1~L4), hip BMD and femoral neck BMD with SI. (D-F) Simple correlation analyses of spine BMD (L1~L4), hip BMD and femoral neck BMD with CCR. SI: sarcopenia index; CCR: creatinine/Cystatin C ratio.

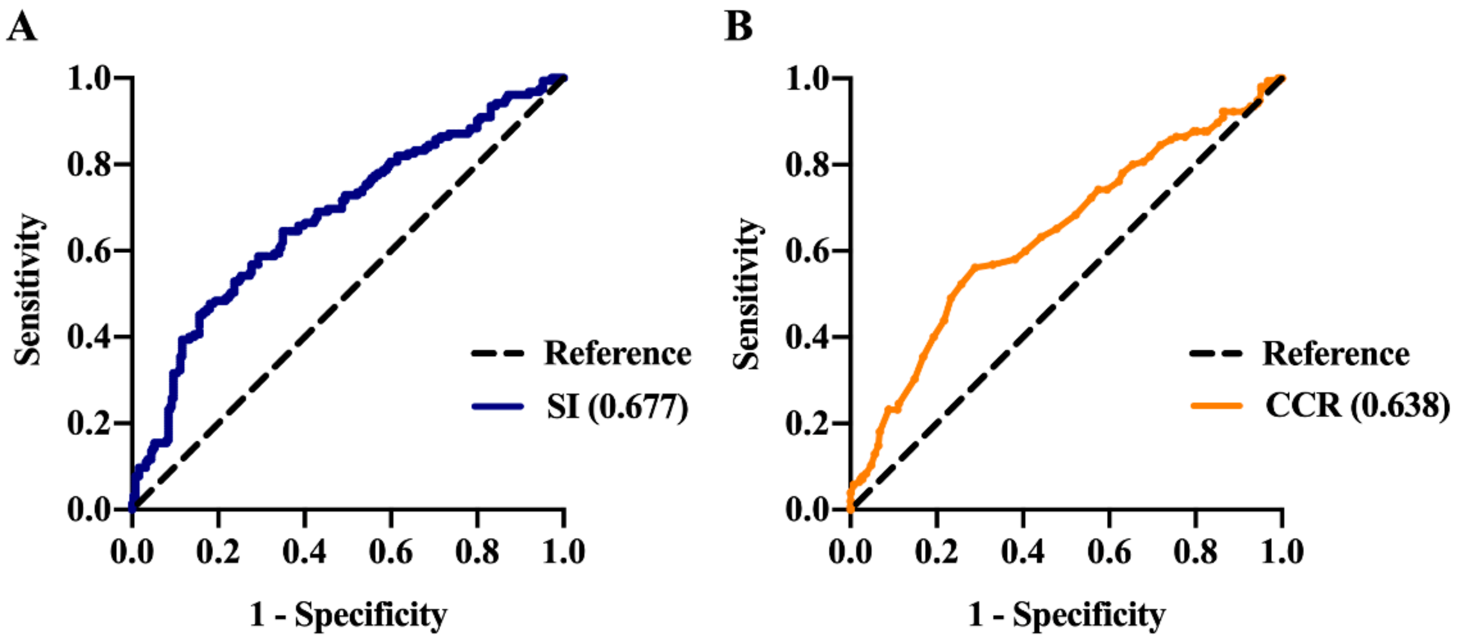

Figure S3. ROC curves of SI (A) and CCR (B) in predicting sarcopenia. Reference is the tracing of ROC analysis of equation in predicting sarcopenia. Abbreviations: ROC: Receiver Operating Characteristic; SI: sarcopenia index; CCR: creatinine/Cystatin C ratio.

Table S1. Multivariable analyses for logistic regression of new fall and new fracture within one year

	Model 1		Model 2		Model 3	
	OR (95%CI)	*P* value	OR (95%CI)	*P* value	OR (95%CI)	*P* value
New fall (N=41) [a]						
CCR (per 1-SD)	0.462(0.293, 0.730)	<0.01**	0.513(0.312, 0.841)	<0.01*	0.507(0.302, 0.849)	0.01*
Age (years)	-	-	1.022(0.967, 1.080)	0.441	1.033(0.977, 1.092)	0.258
BMI (kg/m^2)	-	-	0.749(0.648, 0.866)	<0.001***	0.777(0.666, 0.906)	<0.01**
Sex (female vs. male)	-	-	-	-	1.964(0.744, 5.185)	0.173
Min t-score	-	-	-	-	0.486(0.292, 0.808)	<0.01**
Prior fracture	-	-	-	-	0.255(0.088, 0.740)	0.012*
Prior fall	-	-	-	-	-	-
New fracture (N=37) [b]						
CCR (per 1-SD)	0.470(0.252, 0.876)	<0.05*	0.448(0.229, 0.877)	0.019*	0.510(0.247, 1.053)	0.069
Age (years)	-	-	0.985(0.911, 1.066)	0.71	0.988(0.901, 1.082)	0.791
BMI (kg/m^2)	-	-	0.881(0.740, 1.049)	0.153	0.969(0.804, 1.168)	0.74
Sex (female vs. male)	-	-	-	-	1.216(0.263, 5.628)	0.803
Min t-score	-	-	-	-	0.374(0.190, 0.739)	<0.01**
Prior fall	-	-	-	-	7.742(1.691, 35.447)	<0.01**
Prior fracture	-	-	-	-	-	-

Multivariate logistic regression analyses were performed to determine whether CCR was independently associated with new fall and new fracture within one year. Abbreviations: CCR: creatinine/cystatin C ratio; SD: standard deviation; OR: odds ratio; 95% CI: 95% confidential interval; BMI: body mass index; Min: minimum. Dependent variables: new fracture and new fall; Independent variables: CCR, age, BMI, gender, Min t-score, and history of falls and fractures. [a] Logistic regression to predict fall at 12 months' follow-up in participants without a fall

at baseline (n=378). [b] Logistic regression to predict fracture at 12 months' follow-up in participants without a fracture at baseline (n=293). * $P < 0.05$, ** $P < 0.01$, *** $P < 0.001$.

Table S2. ROCs for SI, CCR and predictive equations as predictors of new fall and new fracture

	Optimized cutoff value	Sensitivity (%)	Specificity (%)	Youden index	AUC	Standard error (SE)	95% CI	*P* value
New fall within one year								
SI	0.085	77.1	50.6	0.278	0.67	0.045	0.582-0.759	<0.001***
Equation (including SI) for new fall	0.078	82.9	66.9	0.497	0.815	0.032	0.753-0.877	<0.001***
CCR	0.111	67.2	62.9	0.300	0.675	0.046	0.586-0.764	<0.001***
Equation (including CCR) for new fall	0.066	62.0	88.6	0.505	0.810	0.033	0.746-0.874	<0.001***
New fracture within one year								
SI	0.035	93.8	34.5	0.282	0.664	0.066	0.535-0.793	0.028*
Equation (including SI) for new fracture	0.083	75	83.7	0.587	0.850	0.047	0.758-0.942	<0.001***
CCR	0.045	42.8	87.5	0.303	0.668	0.065	0.541-0.796	<0.05*
Equation (including CCR) for new fracture	0.094	86.0	75.0	0.700	0.841	0.055	0.734-0.948	<0.001***

Equation (including SI) for new fall: 5.043 - 0.054 × SI – 0.253 × BMI (kg/m_2) – 1.328 × prior fracture (yes=1) - 0.576 × Min t-score. Equation (including SI) for new fracture: -3.312 - 0.048 × SI + 2.009 × prior fall (yes=1) -1.003 × Min t-score. Equation (including CCR) for new fall: 5.360 – 4.639 × CCR - 0.239 × BMI (kg/m_2) - 1.309 × prior fracture (yes=1) - 0.560 × Min t-score. Equation (including CCR) for new fracture: - 2.771 – 4.119 × CCR + 2.055 × prior fall (yes=1) - 0.986 × Min t-score. Abbreviations: ROC: Receiver Operating Characteristic; SI: sarcopenia index; CCR: creatinine/cystatin C ratio; AUC: area under the curve; 95% CI: 95% confidence interval; Min: minimum. * $P < 0.05$; *** $P < 0.001$.
